# (1*H*-Benzimidazol-1-yl)methanol

**DOI:** 10.1107/S1600536812004114

**Published:** 2012-02-04

**Authors:** Augusto Rivera, Mauricio Maldonado, Jaime Ríos-Motta, Karla Fejfarová, Michal Dušek

**Affiliations:** a Departamento de Química, Universidad Nacional de Colombia, Ciudad Universitaria, Bogotá, Colombia; b Institute of Physics ASCR, v.v.i., Na Slovance 2, 182 21 Prague 8, Czech Republic

## Abstract

In the title compound, C_8_H_8_N_2_O, the N—CH_2_ and CH_2_—O bond lengths can be correlated to the manifestation of an anomeric effect in the N—CH_2_—O moiety. In the crystal, inter­molecular O—H⋯N hydrogen bonds link the mol­ecules into zigzag chains, with graph-set motif *C*(6), parallel to [001]. These chains are further linked into sheets by weak nonclassical C—H⋯O hydrogen bonds.

## Related literature

For a related structure, see: Shi *et al.* (2011 ▶). For bond-length data, see: Allen *et al.* (1987 ▶). For chemical background on the synthesis and uses of the title compound, see: Milata *et al.* (2001 ▶). For graph-set analysis, see: Bernstein *et al.* (1995 ▶).
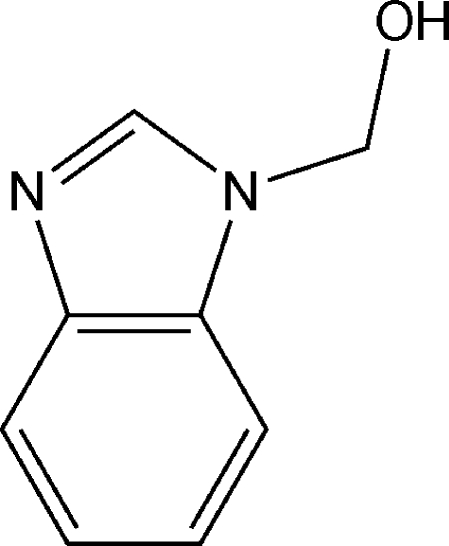


## Experimental

### 

#### Crystal data

C_8_H_8_N_2_O
*M* *_r_* = 148.2
Monoclinic, 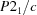

*a* = 13.3181 (10) Å
*b* = 4.2677 (3) Å
*c* = 12.4795 (10) Å
β = 95.143 (6)°
*V* = 706.45 (9) Å^3^
*Z* = 4
Cu *K*α radiation
μ = 0.78 mm^−1^
*T* = 120 K
0.41 × 0.30 × 0.23 mm

#### Data collection

Agilent Xcalibur diffractometer with an Atlas (Gemini Ultra Cu) detector
Absorption correction: multi-scan (*CrysAlis PRO*; Agilent, 2010 ▶) *T*_min_ = 0.744, *T*_max_ = 1
4736 measured reflections
1248 independent reflections
1086 reflections with *I* > 3σ(*I*)
*R*_int_ = 0.032

#### Refinement

*R*[*F* ^2^ > 2σ(*F* ^2^)] = 0.039
*wR*(*F* ^2^) = 0.105
*S* = 1.78
1248 reflections
103 parameters
H atoms treated by a mixture of independent and constrained refinement
Δρ_max_ = 0.18 e Å^−3^
Δρ_min_ = −0.22 e Å^−3^

### 

Data collection: *CrysAlis PRO* (Agilent, 2010 ▶); cell refinement: *CrysAlis PRO*; data reduction: *CrysAlis PRO*; program(s) used to solve structure: *SIR2002* (Burla *et al.*, 2003 ▶); program(s) used to refine structure: *JANA2006* (Petříček *et al.*, 2006 ▶); molecular graphics: *DIAMOND* (Brandenburg & Putz, 2005 ▶); software used to prepare material for publication: *JANA2006*.

## Supplementary Material

Crystal structure: contains datablock(s) global, I. DOI: 10.1107/S1600536812004114/bx2398sup1.cif

Structure factors: contains datablock(s) I. DOI: 10.1107/S1600536812004114/bx2398Isup2.hkl

Supplementary material file. DOI: 10.1107/S1600536812004114/bx2398Isup3.cml

Additional supplementary materials:  crystallographic information; 3D view; checkCIF report

## Figures and Tables

**Table 1:** Hydrogen-bond geometry (Å, °)

*D*—H⋯*A*	*D*—H	H⋯*A*	*D*⋯*A*	*D*—H⋯*A*
O1—H1o⋯N2^i^	0.894 (19)	1.85 (2)	2.7355 (16)	173.8 (17)
C1—H1⋯O1^ii^	0.96	2.41	3.2887 (17)	152

Symmetry codes: (i) 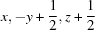; (ii) 
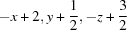
.

**Fig. 1.**
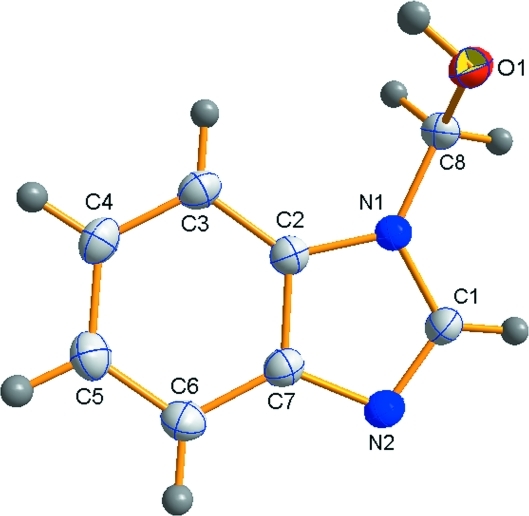
A view of the title molecule. Displacement ellipsoids are drawn at the 50% probability level.

**Fig. 2.**
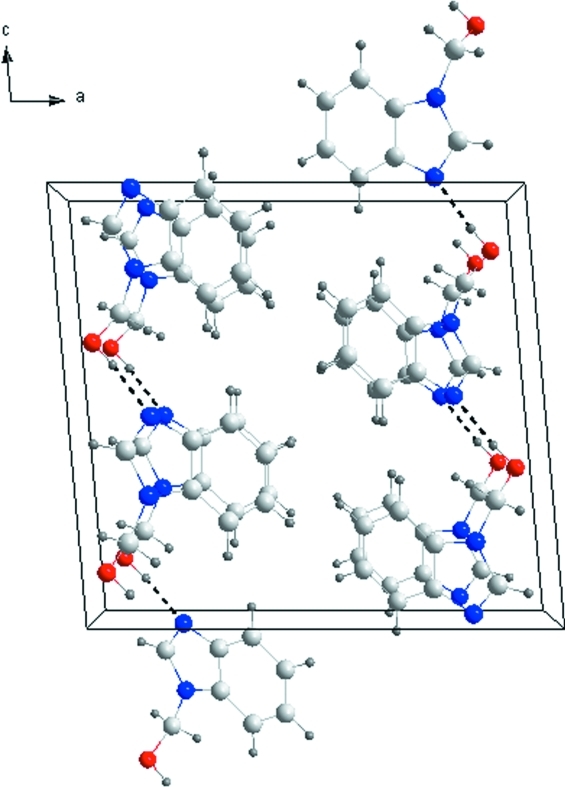
Packing of the molecules of the title compound view along the b axis. Dashed lines indicate the intermolecular hydrogen bonds.

## supplementary crystallographic information

### Comment

Benzimidazole derivatives are compounds that have received much attention because of their applications in several areas. Although the synthesis of title compound had been reported in the literature (Milata *et al.*, 2001), we have developed an alternative route to prepare this compound starting from the synthetically available benzoaminal 6*H*,13*H*-5:12,7:14-dimethanedibenzo[*d,i*][1,3,6,8]tetraazecine (DMDBTA).

In the title compound, C_8_H_8_N_2_O, (Fig.1) the benzimidazole ring is essentially planar, with a maximum deviation for N1 of 0.0089 (12)Å from the least-squares plane defined by the nine constituent atoms. The sum of bond angles around this nitrogen atom was 359.90 (11)°, which is consistent with the planarization of the heterocyclic ring. The distances within the benzimidazole ring of the title compound are very similar to those found in bis(1*H*-benzimidazol-1-yl)methane monohydrate (Shi *et al.*, 2011). However, the observed N—CH_2_ bond length [N1—C8, 1.4638 (17) Å] is longer in relation to the mentioned mean value observed in related structure [N—CH_2_, 1.452 (4) Å] (Shi *et al.*, 2011). Moreover, the CH_2_—O bonds in the residue tend to be shorter than the normal values by 0.033 Å (Allen *et al.*, 1987). This fact can be correlated to the manifestation of an anomeric effect in N—CH_2_—O moiety, but it operates in the opposite direction.In the crystal structure, intermolecular O—H···N hydrogen bonds link the molecules into *zigzag* chains with graph-set motif C(6) parallel to [001], (Bernstein *et al.*, 1995) (Fig. 2). These chains are further linked into sheet by weak non-classical C—H···O hydrogen bonds between H atom of the benzimidazole ring and the O atom of a neighbouring molecule.

### Experimental

A solution of 6*H*,13*H*-5:12,7:14-dimethanedibenzo[*d,i*][1,3,6,8] tetraazecine (DMDBTA) (0.25 mmol) and *p*-nitrophenol (0.5 mmol) in propan-2-ol (15 ml) was placed in a round-bottomed flask equipped with a water-cooled condenser. The reaction mixture was heated at 347 K for 3 h. giving a white precipitate which was filtered off, and the mother liquor was then concentrated by a rotary evaporator to give an oil accompanied by precipitates, which was removed by filtration. Single crystal of the precipitate (title compound), suitable for X-ray crystallography, was grown by slow evaporation from water:propan-2-ol solution at room temperature after several days. Melting point 407 K.

The NMR spectra were acquired at room temperature on a Bruker AMX 400 Avance spectrometer. ^1^H NMR (δ, 400 MHz, CDCl_3_): 5.60, 6.78, 7.23, 7.29, 7.66, 8.28. ^13^C NMR (δ, 100 MHz, CDCl_3_): 67.8, 111.4, 119.8, 122.3, 122.9, 133.7, 144.1, 144.8.

### Refinement

The hydroxyl hydrogen atom was found in difference Fourier maps and its coordinates were refined freely. All other H atoms atoms were positioned geometrically and treated as riding on their parent atoms. The isotropic atomic displacement parameters of hydrogen atoms were evaluated as 1.2×*U*_eq_ of the parent atom.

### Crystal data

**Table:**

C_8_H_8_N_2_O	*F*(000) = 312
*M**_r_* = 148.2	*D*_x_ = 1.393 Mg m^−^^3^
Monoclinic, *P*2_1_/*c*	Cu *K*α radiation, λ = 1.5418 Å
Hall symbol: -P 2ybc	Cell parameters from 2690 reflections
*a* = 13.3181 (10) Å	θ = 3.3–66.8°
*b* = 4.2677 (3) Å	µ = 0.78 mm^−^^1^
*c* = 12.4795 (10) Å	*T* = 120 K
β = 95.143 (6)°	Block, colourless
*V* = 706.45 (9) Å^3^	0.41 × 0.30 × 0.23 mm
*Z* = 4	

### Data collection

**Table:**

Agilent Xcalibur diffractometer with an Atlas (Gemini ultra Cu) detector	1248 independent reflections
Radiation source: Enhance Ultra (Cu) X-ray Source	1086 reflections with *I* > 3σ(*I*)
Mirror monochromator	*R*_int_ = 0.032
Detector resolution: 10.3784 pixels mm^-1^	θ_max_ = 67.1°, θ_min_ = 3.3°
rotation method data acquisition using ω scans	*h* = −15→15
Absorption correction: multi-scan (*CrysAlis PRO*; Agilent, 2010)	*k* = −5→5
*T*_min_ = 0.744, *T*_max_ = 1	*l* = −14→14
4736 measured reflections	

### Refinement

**Table:**

Refinement on *F*^2^	29 constraints
*R*[*F*^2^ > 2σ(*F*^2^)] = 0.039	H atoms treated by a mixture of independent and constrained refinement
*wR*(*F*^2^) = 0.105	Weighting scheme based on measured s.u.'s *w* = 1/[σ^2^(*I*) + 0.0016*I*^2^]
*S* = 1.78	(Δ/σ)_max_ = 0.002
1248 reflections	Δρ_max_ = 0.18 e Å^−^^3^
103 parameters	Δρ_min_ = −0.22 e Å^−^^3^
0 restraints	

### Special details

**Table:**

Experimental. CrysAlisPro (Agilent , 2010) Empirical absorption correction using spherical harmonics, implemented in SCALE3 ABSPACK scaling algorithm.
Refinement. The refinement was carried out against all reflections. The conventional *R*-factor is always based on *F*. The goodness of fit as well as the weighted *R*-factor are based on *F* and *F*^2^ for refinement carried out on *F* and *F*^2^, respectively. The threshold expression is used only for calculating *R*-factors *etc*. and it is not relevant to the choice of reflections for refinement.The program used for refinement, Jana2006, uses the weighting scheme based on the experimental expectations, see _refine_ls_weighting_details, that does not force *S* to be one. Therefore the values of *S* are usually larger than the ones from the *SHELX* program.

### Fractional atomic coordinates and isotropic or equivalent isotropic displacement parameters (Å^2^)

**Table:**

	*x*	*y*	*z*	*U*_iso_*/*U*_eq_	
O1	0.93101 (7)	0.3994 (2)	0.86004 (8)	0.0311 (3)	
N1	0.83140 (8)	0.4446 (3)	0.69371 (9)	0.0247 (3)	
N2	0.81868 (9)	0.2744 (3)	0.52326 (9)	0.0291 (4)	
C1	0.86934 (10)	0.4481 (3)	0.59626 (11)	0.0277 (4)	
C2	0.74835 (9)	0.2487 (3)	0.68347 (10)	0.0243 (4)	
C3	0.68095 (10)	0.1551 (3)	0.75625 (11)	0.0268 (4)	
C4	0.60448 (10)	−0.0444 (3)	0.71773 (11)	0.0289 (4)	
C5	0.59534 (10)	−0.1479 (3)	0.61064 (12)	0.0308 (4)	
C6	0.66286 (10)	−0.0572 (3)	0.53902 (11)	0.0296 (4)	
C7	0.74087 (9)	0.1443 (3)	0.57631 (10)	0.0255 (4)	
C8	0.87514 (9)	0.6034 (3)	0.79091 (10)	0.0271 (4)	
H1	0.927903	0.566907	0.582034	0.0333*	
H3	0.687318	0.225735	0.829577	0.0322*	
H4	0.556364	−0.114071	0.765431	0.0347*	
H5	0.540778	−0.285048	0.586627	0.037*	
H6	0.656443	−0.13025	0.465963	0.0356*	
H8a	0.91753	0.77186	0.771157	0.0325*	
H8b	0.822274	0.695733	0.827835	0.0325*	
H1o	0.8905 (14)	0.341 (4)	0.9098 (15)	0.0373*	

### Atomic displacement parameters (Å^2^)

**Table:**

	*U*^11^	*U*^22^	*U*^33^	*U*^12^	*U*^13^	*U*^23^
O1	0.0258 (5)	0.0439 (6)	0.0239 (5)	0.0053 (4)	0.0035 (4)	0.0043 (4)
N1	0.0236 (6)	0.0287 (6)	0.0218 (6)	−0.0009 (4)	0.0024 (4)	−0.0001 (4)
N2	0.0285 (6)	0.0358 (7)	0.0235 (6)	−0.0006 (4)	0.0053 (4)	−0.0005 (5)
C1	0.0253 (7)	0.0338 (8)	0.0248 (7)	−0.0016 (5)	0.0056 (5)	0.0012 (5)
C2	0.0235 (6)	0.0249 (7)	0.0244 (7)	0.0038 (5)	0.0017 (5)	0.0013 (5)
C3	0.0272 (7)	0.0287 (7)	0.0250 (7)	0.0048 (5)	0.0052 (5)	0.0014 (5)
C4	0.0252 (7)	0.0297 (7)	0.0327 (8)	0.0028 (5)	0.0070 (6)	0.0055 (5)
C5	0.0261 (7)	0.0300 (7)	0.0355 (8)	−0.0017 (5)	−0.0014 (6)	0.0024 (6)
C6	0.0323 (7)	0.0305 (7)	0.0255 (7)	0.0005 (5)	−0.0008 (6)	−0.0002 (5)
C7	0.0254 (7)	0.0267 (7)	0.0243 (7)	0.0030 (5)	0.0029 (5)	0.0008 (5)
C8	0.0268 (7)	0.0309 (7)	0.0234 (7)	−0.0002 (5)	0.0015 (5)	−0.0010 (5)

### Geometric parameters (Å, º)

**Table:**

O1—C8	1.3932 (16)	C3—C4	1.3804 (18)
O1—H1o	0.894 (19)	C3—H3	0.96
N1—C1	1.3581 (18)	C4—C5	1.402 (2)
N1—C2	1.3834 (16)	C4—H4	0.96
N1—C8	1.4638 (17)	C5—C6	1.379 (2)
N2—C1	1.3133 (17)	C5—H5	0.96
N2—C7	1.3941 (18)	C6—C7	1.3961 (18)
C1—H1	0.96	C6—H6	0.96
C2—C3	1.3916 (19)	C8—H8a	0.96
C2—C7	1.4045 (18)	C8—H8b	0.96
			
C8—O1—H1o	106.4 (11)	C5—C4—H4	119.1913
C1—N1—C2	106.41 (11)	C4—C5—C6	121.57 (12)
C1—N1—C8	125.77 (11)	C4—C5—H5	119.2139
C2—N1—C8	127.72 (11)	C6—C5—H5	119.2142
C1—N2—C7	104.65 (11)	C5—C6—C7	117.76 (13)
N1—C1—N2	113.94 (12)	C5—C6—H6	121.1194
N1—C1—H1	123.0293	C7—C6—H6	121.1187
N2—C1—H1	123.0296	N2—C7—C2	109.49 (11)
N1—C2—C3	132.10 (12)	N2—C7—C6	130.52 (12)
N1—C2—C7	105.52 (11)	C2—C7—C6	119.99 (12)
C3—C2—C7	122.38 (11)	O1—C8—N1	112.01 (11)
C2—C3—C4	116.66 (12)	O1—C8—H8a	109.4718
C2—C3—H3	121.6681	O1—C8—H8b	109.4708
C4—C3—H3	121.6689	N1—C8—H8a	109.4717
C3—C4—C5	121.62 (13)	N1—C8—H8b	109.4709
C3—C4—H4	119.1906	H8a—C8—H8b	106.8062

### Hydrogen-bond geometry (Å, º)

**Table:**

*D*—H···*A*	*D*—H	H···*A*	*D*···*A*	*D*—H···*A*
O1—H1*o*···N2^i^	0.894 (19)	1.85 (2)	2.7355 (16)	173.8 (17)
C1—H1···O1^ii^	0.96	2.41	3.2887 (17)	152

Symmetry codes: (i) *x*, −*y*+1/2, *z*+1/2; (ii) −*x*+2, *y*+1/2, −*z*+3/2.
